# Comparison of three different methods to detect bone marrow involvement in patients with neuroblastoma

**DOI:** 10.1007/s00432-021-03780-7

**Published:** 2021-10-08

**Authors:** Felix Schriegel, Sabine Taschner-Mandl, Marie Bernkopf, Uwe Grunwald, Nikolai Siebert, Peter F. Ambros, Inge Ambros, Holger N. Lode, Guenter Henze, Karoline Ehlert

**Affiliations:** 1 Department of Pediatric Oncology and Hematology, University Medicine Greifswald, Ferdinand-Sauerbruch-Strasse, D-17475 Greifswald, Germany; 2 Department of Medicine C, Hematology and Oncology, University Medicine Greifswald, Greifswald, Germany; 3 CCRI, St. Anna Children’s Cancer Research Institute, Vienna, Austria; 4 Department of Pediatric Oncology and Hematology, Charité University Medicine Berlin, Campus Virchow Klinikum, Berlin, Germany

**Keywords:** Neuroblastoma, Bone marrow, Survival, Minimal residual disease

## Abstract

**Purpose:**

Neuroblastoma (NB) is the most frequent extracranial tumor in children. The detection of bone marrow (BM) involvement is crucial for correct staging and risk-adapted treatment. We compared three methods regarding the detection of NB involvement in BM.

**Methods:**

Eighty-one patients with NB were included in this retrospective study. BM samples were obtained at designated time points at study entry and during treatment or follow-up. The diagnostic tools for BM analysis included cytomorphology (CM), flow cytometry (FCM) and automatic immunofluorescence plus fluorescence in situ hybridization (AIPF).

**Results:**

We analyzed 369 aspirates in 81 patients in whom AIPF, CM, and FCM were simultaneously available. During the observation period, NB cells were detected in 86/369 (23.3%) cases, by CM in 32/369 (8.7%), by FCM in 52 (14.1%), and by AIPF in 72 (19.5%) samples. AIPF and/or FCM confirmed all positive results obtained in CM and detected 11 additional positive BM aspirates in 294 CM negative samples (*p* < 0,001). Survival of patients with BM involvement at study entry identified solely by FCM/AIPF was 17.4% versus 0% for patients in whom BM involvement was already identified by CM.

**Conclusion:**

The combination of AIPF/FCM yielded the highest detection rate of NB cells in BM. AIPF was the single, most sensitive method in detecting these cells. Although CM did not provide any additional positive results, it is still a useful, readily available and cost-effective tool. The prognostic significance of FCM and AIPF should be confirmed in a prospective study with a larger number of patients.

## Introduction

Neuroblastoma (NB) is the most frequent solid malignancy of childhood outside the central nervous system and represents a biologically heterogeneous group of embryonic tumors arising either from the adrenal gland or primitive sympathetic ganglion cells. The highest incidence is observed in children below the age of 4 years (Schleiermacher et al. 2014; Brodeur and Bagatell 2014; Matthay et al. 2016).

Precise knowledge about the tumor´s characteristics and dissemination is crucial for risk-adapted treatment (Terzic et al. 2017). Neuroblastoma often presents with multilocular bone marrow (BM) involvement that may be difficult to detect as the amount of BM infiltrating tumor cells is usually below 30%, even at diagnosis (Abbasi et al. 2015). Different methods for assessing BM involvement are available either by analyzing the tumor cells by morphological or immunocytological techniques or indirectly, for instance by quantification of NB specific RNA molecules (Beiske et al. 2009; Morandi et al. 2015). Cytomorphological evaluation by light microscopy offers the advantage of being rapidly available and cost-efficient. Obtaining two BM aspirates and two trephine biopsies is currently considered the standard procedure (Burchill et al. 2017), as former studies have demonstrated that the combination of these two methods has yielded the highest rate of detection of NB cells in BM (Franklin and Pritchard 1983; Aronica et al. 1998). However, gaining adequate trephine biopsies particularly in small children can be difficult (Reid and Roald 1999; Morotti et al. 2016; Burchill et al. 2017). In the past few years, immunologically based methods for assessing BM involvement in NB have increased the spectrum of diagnostic tools (Ambros et al. 2003; Morandi et al. 2015; Druy et al. 2018; Popov et al. 2019).

In this retrospective single-center cohort study in a large German pediatric institution specialized in NB research and treatment, three different methods of BM investigation were compared to identify the most sensitive set of tools for the detection of NB in BM.

## Materials and methods

### Study population

From July, 2009, until April, 2015, 81 patients with NB were referred to the pediatric oncology department at the University Medical Center Greifswald. All patients were to receive either i) immune therapy with dinutuximab beta or ii) salvage chemotherapy after standard first-line or relapse treatment for NB according to the respective national or international protocols or iii) both treatment approaches. Patients were excluded from the analyses if during the observation period BM samples were not consistently investigated by cytomorphology (CM), flow cytometry (FCM) and automated immunofluorescence plus FISH (AIPF).

Diagnostic workups were performed before study entry and repeated every 3–4 months at defined time points according to the respective trial protocol. Each diagnostic procedure included assessment of BM involvement with different methods and imaging studies as outlined below.

The patient disease stage and risk group at diagnosis was determined according to the International Neuroblastoma Staging System (INSS) and the International Risk Group (INRG) classification system (Brodeur et al. 1993; Cohn et al. 2009; Tolbert and Matthay 2018; Sokol and Desai 2019). According to the results of the initial diagnostic procedure, patients were either defined to be in complete remission or with evidence of neuroblastoma disease. Considering survival, the most recent data were collected on September 30, 2020.

For each patient, informed consent was obtained from parents or guardians.

### Diagnostic procedures

#### ^***123***^***I-mIBG-scintigraphy***

^123^I-mIBG-scans were performed according to the guidelines of the European Association of Nuclear Medicine (EANM). The isotope iodine 123 (^123^I) was applied according to the dosage card version 01.05.2008 from the EANM. The scans were reviewed by senior physicians at the department of nuclear medicine at University Medicine Greifswald.

### Magnetic resonance imaging (MRI) with and without gadolinium

Two different MR scanners, the Siemens MAGNETOM Aera 1.5 Tesla and the Siemens MAGNETOM Symphony 1.5 Tesla were used. Experienced pediatric radiologists reviewed the MRI scans. All scans were presented and discussed in institutional tumor board meetings.

### Bone marrow assessment

Due to the heterogeneous distribution of NB cells in the BM, material from at least two different sites of the pelvic bone (mostly posterior iliac crest) was obtained under general anesthesia. BM involvement was regarded as positive if at least one of the methods described below revealed the presence of NB cells. Except for AIPF (CCRI, St. Anna Children’s Cancer Research Institute, Labdia, Labordiagnostik GmbH, Vienna, Austria), all diagnostic tests were performed in specialized laboratories and departments at University Medicine Greifswald, Germany.

### Cytomorphology of BM smears by light microscopy

At least 10 smears were obtained from each puncture site. Bone marrow was spread out on slides and prepared with *Pappenheim*`s staining procedure. In search of single NB cells or tumor nests, 2 × 200 cells were counted on an Olympus BX53 microscope. An experienced and licensed medical-technical assistant examined all slides. Findings were reviewed by at least one pediatric oncologist. The pediatric hematologic-oncologic laboratory subjects itself to yearly quality assurance procedures.

### Flow cytometry (FCM)

After aspiration BM samples were stained with monoclonal murine antibodies. FCM analyses were performed on a Bioscience BD FCM Calibur dual laser. In each sample, 2—2.5 × 10^5^ events were analyzed. The analyzing software was BD CellQuest Pro and WinList 3.2. Gated cells were positive for CD56 and disialoganglioside GD_2_ and negative for CD3 and CD45 (Beiske et al. 2005). Neuroblastoma BM involvement was regarded to be present if > / = 0.01% among all nucleated cells were CD56/GD_2_ positive. The FCM laboratory takes part in yearly routine quality management procedures.

### Automatic immunofluorescence plus FISH (AIPF)

Cytospin preparations of BM mononuclear cells (MNC) were subjected to AIPF, a combined immunocytologic/cytogenetic method to detect minimal residual disease (MRD). The immunocytological and cytogenetic method of Ambros and Méhes is using GD_2_ and CD56 antibodies to bind to tumor cells, which is visualized and quantified using an automated slide scanning microscope and the RC-Detect Software (both by Metasystems) (Méhes et al. 2001, 2003; Ambros et al. 2003). In cases with less than three GD_2_/CD56 positive cells per 1 × 10^6^ mononuclear cells (MNC) detected or in cases with unclear results, the suspicious cells were scrutinized with interphase Fluorescent in Situ Hybridization (iFISH).

Besides detecting CD56/GD_2_ positive cells with a sensitivity of one tumor cell per 1 × 10^6^ MNCs, the presence of e.g. amplified *MYCN* copies, 17q gain and aneusomies of chromosomes or chromosome arms can be detected to verify immunofluorescence positive cells. More than 10^6^ nucleated cells can be analyzed in one sample (Beiske et al. 2005). The analysis of 3 × 10^6^ MNCs is recommended (Beiske et al. 2005). Samples from different aspiration sites were analyzed separately. AIPF results are available within 48 h after sampling. The laboratory is certified by the International Organization for Standardization, ISO 9001.

### Tumor board meetings

The patient disease status and further diagnostic and therapeutic measures were discussed at weekly, interdisciplinary tumor board meetings.

### Statistics

For statistical analysis, Pearson´s Chi-squared test was conducted to compare the results of the different methods for BM assessment. *P* values were adjusted by the Benjamini–Hochberg method (Benjamini et al. 2017; Jafari and Ansari-Pour 2019) to correct for multiple testing. *P* values lower than 0.05 were considered statistically significant. Every plausible combination of BM study was analyzed through cross-tabulations. For statistical analysis, we used GraphPad software QuickCalcs, 2018 version, and R, 3.6.3 version. Survival of the patients was related to the overall status of disease and particularly to the results of BM involvement at study entry.

## Results

### Patient characteristics

Between July 2009, and April 2015, 81 patients with NB fulfilling the criteria mentioned above were treated at our institution. Sixty-two received anti-GD_2_ based immune therapy with dinutuximab beta, three patients received salvage chemotherapy and 16 patients had treatment with both dinutuximab beta and chemotherapy. At diagnosis, 72 patients (88.9%) were classified as high-risk and six (7.4%) as intermediate risk according to the INRG classification system. In three patients the INRG system was not applicable due to missing data. Stage 4 NB was diagnosed in 73 (90.1%) and stage 3 NB in eight (9.9%) patients. *MYCN* amplification was present in 25 (30.9%) and absent in 44 (54.3%) tumors. The *MYCN* status was unknown in twelve (14.8%) tumors. At the start of the observation period, 18 (22.2%) patients were in complete remission and 63 (77.8%) patients had measurable disease (Table 1).

**Table 1:** Patient characteristics (*N* = 81)

	*N *(%)
Gender	
Male	49 (60.5)
Female	32(39.5)
Age (years)	
Median	6.6
Range	1.1–24.5
INSS at diagnosis	
Stage 3	8(9.9)
Stage 4	73(90.1)
INRG classification at diagnosis	
High-risk	72(88.9)
Intermediate risk	6(7.4)
Unknown	3(3.7)
MYCN	
Amplified	25(30.9)
Non-amplified	44(54.3)
Unknown	12(14.8)
Remission status at study entry	
Complete remission	18(22.2)
Measurable disease	63(77.8)
Complete number of bone marrow assessments	
Total aspirates	369
Number of imaging studies	
MRI	354
mIBG	361

*INSS* international neuroblastoma staging system, *INRG* international neuroblastoma risk group, *FCM* flow cytometry, *AIPF* automatic immunofluorescence plus FISH, *MRI* magnetic resonance imaging, *mIBG* metaiodobenzylguanidine

For analysis, 369 BM aspirates were obtained and analyzed with CM, FCM and AIPF. Due to the limited number of trephine biopsies, these were excluded from statistical analyses.

The evaluation procedures also included MRI and scintigraphy. In total, 361 mIBG and 354 MRI scans were performed (Table 1).

### Overall BM involvement in NB

**Fig. 1:**
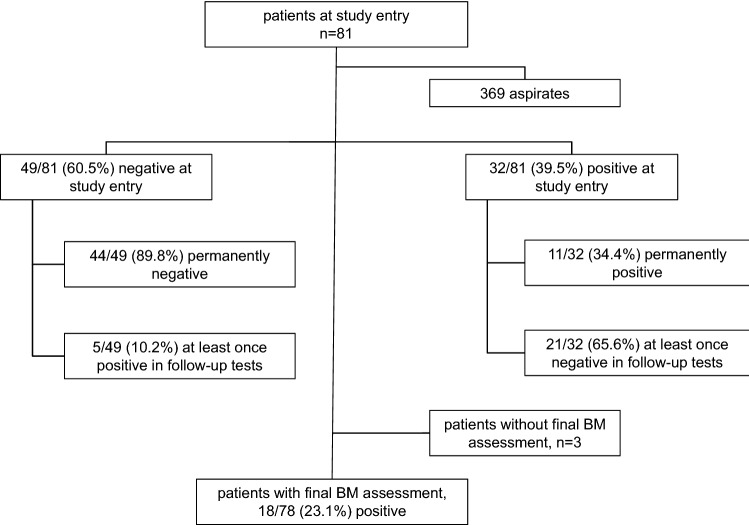
Flow-chart: bone marrow involvement at study entry and during follow-up

At the patients´ first BM assessment, NB cells were found in 32/81 (39.5%) aspirates. During the observation period in our institution, four different conditions regarding BM involvement were identified: (i) five patients with initially negative BM who occasionally developed BM involvement; (ii) 44 patients who never presented with NB cells in BM; (iii) 11 patients who were permanently positive for NB cells in BM, and (iv) 21 patients in whom NB cells in BM were absent in some, but not in all assessment time points during treatment (Fig. 1). At the patients´ final assessment, NB BM involvement was proven in 18/78 (23.1%) patients. Sixteen of these 18 patients did not survive. Three patients did not have a final BM assessment.

### Analysis of BM involvement with three different methods

Bone marrow involvement in 369 aspirates was detected by CM in 32 (8.7%) samples, by FCM in 52 (14.1%) samples, and by AIPF in 72 (19.5%) samples.

In each BM aspirate, 400 cells were reviewed by light microscopy. BM samples with reduced cellularity were found in 37.4%. In 22/32 CM positive samples, more than 1% NB cells were detected. The highest single rate of BM infiltration in a sample was 90%. Cytomorphology was reviewed as negative in 91.3%.

By flow cytometry, 2–2.5 × 10^5^ cells were analyzed in each sample. In 52 positive BM samples, the average rate of NB cells was 1.28% (range 0.01–28.3%, median 0.045%).

The mean number of cells analyzed by AIPF was 4.26 × 10^6^ (range 0.22–9.78, median 4.12). In 72 NB positive samples, the average number of analyzed cells was 3.3 × 10^6^ (range 0.22–9.78, median 2.88). The mean NB cell content in the positive samples was 6.29% (range 0.000012–94.5%, median 0.0067%).

### Comparison of FCM with AIPF

Results of FCM and AIPF were available at the same time points in 369 BM samples. Neuroblastoma cells were identified in 86 (23.3%) of these aspirates. FCM and AIPF were simultaneously positive in 38/86 (44.2%) samples. In 34 samples, only AIPF revealed a positive result, in 14 samples only FCM.

When NB cells in BM were detected by FCM, according results with AIPF were obtained in 38/52 samples (73.1%). In 12/14 differing results, the degree of BM infiltration was 0.01% or less.

When NB cells in BM were detected by AIPF, according results with FCM were obtained in 38/72 samples (52.8%). In 34 differing results, the degree of BM infiltration was even lower and on average 0.0028% (median 0.00039%).

### Cytomorphology compared to FCM and AIPF

With CM, 337/369 (91.3%) of samples were rated negative. Of these 369 samples, AIPF was negative in 297 (80.5%) and FCM in 317 (86%) samples. Positive results with CM were consistently confirmed by AIPF and/or FCM. In addition, AIPF or FCM identified 54 additional cases of sub-microscopic BM infestation in these 337 cytomorphological negative samples.

In 321 samples, results of CM were compared with the results of the immunological methods FCM and AIPF, when these were either both negative or both positive. FCM and AIPF confirmed all 27 CM positive samples and detected eleven additional BM aspirates with NB cells. In those eleven samples, the degree of BM infiltration was low with a median of 0.02% and a mean of 0.11%. In the 27 positive samples, BM infiltration was higher with a median of 0.5%. Cytomorphology was never positive, when FCM and AIPF were negative. These results were statistically significant (Table 2).

**Table 2:** Comparison of BM assessment methods

	Cytomorphology +	Cytomorphology −	∑
FCM+ /AIPF+	27	11	38
FCM−/AIPF−	0	283	283
∑	27	294	321
	Pearson’s chi-squared test: Χ^2^ = 220, df = 1, *p* < 0.001		

*AIPF* automatic immunofluorescence plus FISH, *FCM* flow cytometry, +  presence of neuroblastoma cells in bone marrow with this method, −  absence of neuroblastoma cells in bone marrow with this method, *df* degrees of freedom, *p* p value from Pearson’s chi-squared test

### Association of MRI/mIBG with BM studies

There were 369 assessment time points with either mIBG or MRI in combination with BM aspirates. In 136/443 (36.9%), there was no evidence of NB using all diagnostic tools. In 194 MRI and/or mIBG scans with evidence of disease, CM did not contribute additional information as FCM and/or AIPF confirmed all positive results obtained by CM. Furthermore, NB cells were identified by FCM and AIPF in 11 additional CM negative BM samples. These results were statistically significant (Table 3). MRI and mIBG yielded no evidence of disease in 143 assessments. In 7 of these samples, NB cells were identified in BM either by FCM (4), or by AIPF (3) but not by CM.

**Table 3:** MRI and mIBG in relation to bone marrow assessment with CM, AIPF, and FCM

MRI / mIBG positive			
	Cytomorphology +	Cytomorphology −	∑
FCM + /AIPF +	27	11	38
FCM −/AIPF−	0	147	147
∑	27	158	185
	Pearson’s Chi-squared test: Χ^2^ = 122, df = 1, *p* < 0.001		

*AIPF* automatic immunofluorescence plus FISH, *FCM* flow cytometry, *mIBG* ^123^I-metaiodobenzylguanidine scintigraphy, *MRI* magnetic resonance imaging, +  presence of neuroblastoma cells in bone marrow with this method,  −  absence of neuroblastoma cells in bone marrow with this method, *df* degrees of freedom, *p* unadjusted p value from Pearson’s Chi-squared test

### Survival

Data on survival were available for 76 patients. Forty-six of 81 (56.8%) patients died of NB, 30 (37%) former study patients are alive with and without residual disease. In five patients from abroad, the current status remains unknown.

All eleven patients with NB cell involvement in the BM during the entire study period did not survive. Nine of nine patients did not survive when NB cells in BM were initially detected by CM. In 23 patients, BM involvement at study entry was only identified by FCM or AIPF. Of these, four (17.4%) are alive, 18 (78.3%) did not survive, and one patient was lost to follow-up.

## Discussion

Bone marrow involvement in children with NB occurs in about 50%. However, in patients with high-risk or metastatic disease BM involvement may be detected in up to 80% of patients (Tolbert and Matthay 2018). Using highly sensitive techniques, BM involvement at diagnosis was found in even more than 90% of stage 4/M patients (Méhes et al. 2001). Earlier studies compared the use of BM aspirates with trephine biopsies and concluded that the highest yield of detection was reached with the combination of both procedures and that BM trephines are slightly more sensitive than aspirates (Favrot et al. 1986; Mills and Bird 1986; Aronica et al. 1998). The German trial NB 2004 recommended performing either four aspirates from four different sites or two aspirates plus two trephine biopsies in case the aspirates did not appear to be representative (Berthold 2004). In the past two decades, immunologically based methods for assessing BM involvement in NB have widened the spectrum of diagnostic tools (Ambros et al. 2003; Méhes et al. 2003; Beiske et al. 2005; Morandi et al. 2015; Druy et al. 2018; Popov et al. 2019).

In this retrospective, single-center study in one of the largest NB research and treatment institutions in Germany, we compared the results of three different methods for the detection of NB cells in BM in 81 patients. These included CM, FCM and AIPF. This combination of BM analysis has been the standard approach in our institution for more than ten years.

BM cellularity was reduced in 37.4% of the aspirates. This is likely due to the fact that all patients had undergone numerous, previous therapies before treatment in our institution. Nevertheless, a high number of cells were available for analysis. NB cells in BM were most often detected by AIPF (19.5%), followed by FCM (14.1%) and CM (8.7%). During the observation period, the number of patients with BM involvement decreased from 39.5% to 23.1%.

BM infiltration detected by FCM was in a range of 0.01–28.3% (median 0.045%) and by AIPF in a range of 0.000012–94.5% (median 0.0067%) and thus in most samples in a sub-microscopic level of detection. This is supported by the finding that FCM and AIPF were able to identify additional BM samples with NB in aspirates which were unremarkable in light microscopy.

Staining of BM smears for CM is a simple procedure that is well established and widely available (Méhes et al. 2001). However, the microscopic investigation of BM smears is time-consuming, and the interpretation depends on the quality and representativeness of the sample, which may be limited in patients who have been heavily pretreated with chemotherapy (Méhes et al. 2003; Burchill et al. 2017; Popov et al. 2019). Another critical point is the examiner's experience, which may be less outside oncology centers than in experienced laboratories (Reid and Roald 1996). In addition, CM is only able to detect NB cells when BM infiltration exceeds the submicroscopic level (Cheung et al. 1997). Here, we did not systematically re-evaluate all CM-negative BM samples if they were found positive by AIPF, as the samples had already primarily been assessed by two independent, experienced BM morphologists. However, CM negativity was confirmed when selected AIPF-positive samples were re-examined by light microscopy.

FCM and AIPF did not show consistent results in each sample. Inconsistencies were particularly found when the degree of BM infiltration was extremely low in a range of 0.01% or less. As a single method, AIPF identified NB cells most frequently and the combination of AIPF and FCM had the highest yield of detection of NB cells in BM.

In 143 assessments with unremarkable results in MRI and mIBG, we identified only seven BM samples with NB cells (4.9%), and those in fact with FCM and AIPF. In patients, whose tumor treatment has been finished for more than two years, our current algorithm is therefore obtaining BM samples only if there is detectable disease in mIBG/MRI. When the imaging studies reveal unremarkable results, we desist from further invasive diagnostics and procedures involving radiation exposure.

The mortality in the study population was 56.8%. The presence of NB cells in BM identified by CM at study entry reflected a more advanced stage of disease with a poor prognosis. All of these patients did not survive. Mortality was also very high in patients with BM involvement detected only by FCM or AIPF (78.3%) which underlines the importance of these immunological methods.

Retrospective analyses carry several limitations. In recent years, molecular diagnostic tests have also been used to assess the infiltration of NB cells into BM. In 2009, the consensus criteria of the International Neuroblastoma Risk Group Task Force for the detection of NB cells in BM were published (Beiske et al. 2009). Immunocytology for GD_2_ and reverse transcriptase quantitative polymerase chain reaction (RTqPCR) for tyrosine hydroxylase were recommended. In 2017, the International Neuroblastoma Response Criteria Bone Marrow Working Group recommended the collection of both bilateral BM biopsies and aspirates and their analysis with immunohistochemistry for GD_2_ and RTqPCR for tyrosine hydroxylase and paired-like homeobox 2B (PHOX2B) (Burchill et al. 2017). Although there may be substantial agreement that biopsies should be added to analysis of BM aspirates, this statement relates to data obtained in the 1980s and 1990s when diagnostic tools to detect minimal disease in aspirates were not well established (Franklin and Pritchard 1983; Verdeguer et al. 1988; Ganick et al. 1988; Thiesse et al. 1991; Hedborg et al. 1992; Brodeur et al. 1993; Monclair et al. 2009). Bone marrow assessment with RTqPCR as recommended by these two guidelines is currently not common clinical practice in Germany. Therefore, a comparison between those approaches and the results of bone marrow assessment analyzed here is not yet possible.

An association of RTqPCR for three different parameters (tyrosine hydroxylase, PHOX2B, doublecortin) in BM at diagnosis with poor outcome was shown (Stutterheim et al. 2008; Viprey et al. 2014). However, the authors also stated that the results of trephine biopsies did not contribute additional information. Its sensitivity is limited and cytology and histology cannot demonstrate sub-microscopic involvement of the BM (Cheung et al. 1997; Stutterheim et al. 2008). Due to the limited number of trephine biopsies obtained in our patients, these were excluded from further analysis.

In summary, AIPF proved to be the single most sensitive method for the detection of BM involvement in NB. The presence of NB cells in BM only detected by AIPF or FACS was able to identify a high-risk population with a poor survival of only 17.4%. Since these are the results of a retrospective study, the diagnostic value and the prognostic significance of FCM and AIPF should be confirmed in a prospective study with a larger number of patients.

## Conclusion

The combined use of AIPF and FCM in BM aspirates resulted in the highest detection rate of NB cells. Cytomorphology has the advantage of being quickly available and inexpensive and should therefore not be abandoned. Given these results, the current procedures for assessing the involvement of BM in patients with NB may safely be reduced to CM, FCM, and AIPF. Further studies are needed to clarify the comparative role of AIPF/FCM and PCR-based methods in assessing BM involvement in patients with NB and its relevance to disease control and outcome.

## Data Availability

The data that support the findings of this study are available from the corresponding author upon request.
